# Assessment of the knowledge of Brazilian Community Health Workers regarding prenatal care

**DOI:** 10.1017/S1463423618000725

**Published:** 2018-10-09

**Authors:** Lívia P. Bonifácio, João M. A. Marques, Elisabeth M. Vieira

**Affiliations:** Social Medicine Department, Ribeirão Preto Medical School, University of São Paulo FMRP/USP, Brazil

**Keywords:** community health agent, community health workers, knowledge, prenatal care, primary healthcare

## Abstract

**Aim:**

To evaluate prenatal care knowledge of Brazilian community health workers (CHWs).

**Background:**

Routine prenatal care is critical for reducing health risks in women and their children. One of the responsibilities of primary healthcare providers is the provision of prenatal care. The CHWs, with their frequent contact with populations, work to improve health outreach efforts and therefore may be key role players in prenatal care.

**Methods:**

This was a cross-sectional study. A questionnaire was developed to ascertain the degree of knowledge regarding prenatal care of CHWs, including: (1) general responsibilities of CHWs; (2) the initial contact with a pregnant woman; (3) examinations and vaccinations recommended for pregnant women; (4) pregnancy complications and signs of labor; and (5) lifestyle considerations for pregnant women. Responses were categorized into levels for knowledge. Demographic data were also collected. Descriptive analyses were conducted. Proportions of subjects with different levels of knowledge were compared according to each demographic variable, separately for each block of knowledge, using the *χ* ^2^ and Fisher’s exact tests.

**Findings:**

In total, 194 CHWs were interviewed. Overall, the majority of the CHWs presented high levels of knowledge in blocks 1 (43%), 2 (59%) and 5 (83%). However, in block 3 the proportions of subjects with high levels of knowledge regarding examinations and vaccinations were 35 and 40%, respectively. Only 24% of the participants presented a high level of knowledge in block 4. Stratified data analyses suggest that females were statistically more likely to have high levels of knowledge, whereas no statistically significant differences were found for the other demographic variables. Health services are already routinely using the questionnaire.

**Conclusion:**

The results suggest that CHWs, especially female CHWs, have an important role in assisting pregnant women in the community. The study indicates the areas of knowledge that require more specific attention from training providers.

## Background

Internationally, since the Alma-Ata Conference in 1978, primary healthcare (PHC) has been recognized as a viable way of meeting the health needs of people, including those more vulnerable, considering the influence of the diverse social determinants on health [World Health Organization (WHO) and UNICEF, 1978; WHO, 1979; WHO, 2008; Labonté *et al*., 2014). The positive results of PHC have been recognized (Labonté *et al*., 2014; Lassi *et al*., 2014) and through it several countries have been investing in the reformulation and reorientation of their health systems in order to achieve quality in care, increase access, promote equity and achieve effectiveness (Bhatia and Rifkin, 2013).

One of the aims of the Millennium Development Goals (MDG), introduced by the United Nations in 2000 and adopted by 191 countries, was to improve maternal health, aiming to reduce maternal mortality rates by 75% (Souza, 2013; United Nations, 2015a). This goal has led to recommendations regarding the implementation of policies to improve prenatal care (Cook and Galli Bevilacqua, 2004; Victora *et al*., 2011).

Given its characteristics, PHC can provide adequate support for pregnant women and community health workers (CHW), as one of its key players, can help to empower them for better quality self-care (Lassi *et al*., 2014). The CHW is a health provider working inside and with the community, encouraging people to take care of their health, since they have a cultural identity and bond with the community (Perry *et al*., 2014). They facilitate the access to the health system, conduct home visits, identify at risk and vulnerable people in specific areas, and develop actions aimed at the welfare of these people, including other social sectors (Lassi *et al*., 2014; Perry *et al*., 2014; Macinko and Harris, 2015).

The CHW programs within PHC around the world have focused on the MDG (Perry *et al*., 2014). Evidence has been presented suggesting that the work of the CHW can reduce maternal mortality and improve access to family planning (Perry *et al*., 2014). Visits by CHWs were found to be associated with improved prenatal care, increased coverage of the tetanus vaccination, increased breastfeeding in the first hours of life and higher levels of knowledge regarding hygiene of the umbilical stump (Lassi *et al*., 2014).

These findings show the importance of adequate CHW training, so that they can recognize the main health problems and are able to reduce maternal mortality and morbidity rates, especially in places with limited resources, such as low- and middle-income countries (Mangham-Jefferies *et al*., 2014). However, it has been reported that challenges exist with regard to establishing adequate criteria for recruiting, training and supervising CHWs, defining their assignments and cost effectiveness due to inadequate standard criteria of professional training, insufficient remuneration or incentives for the CHWs, and lack of supervision and logistical support (Lassi *et al*., 2014; Perry *et al*., 2014).

In Brazil, the National Health System (*Sistema Único de Saúde* – SUS), was instituted in 1988, with the PHC constituting an essential component in its organization (Paim *et al*., 2011; Ramírez *et al*., 2016). It is provided by health units located close to the citizen’s home that are responsible for a geographically delimited population (Paim *et al*., 2011; Moreira & O’Dwyer, 2013; Ramírez *et al*., 2016).

There are two models of organization of PHC units in Brazil. There is an older, traditional public PHC care model, also called the Basic Health Unit (*Unidade Básica de Saúde* – UBS), which has a team composed of at least one internal medicine physician, one gynecologist and one pediatrician, as well as nurses and nursing assistants, a dentist and a dental assistant, which may or may not employ CHWs. The unit can be responsible for a population of up to 18 000 people. The other model is the health units with Family Health Strategy (FHS), which is composed of at least one general physician, one primary care nurse, one nursing assistant and a sufficient number of CHWs capable of covering 100% of the population. The unit is responsible for a population of up to 4000 people (Brazil, 2012; Macinko and Harris, 2015). According to the Brazilian Ministerial Order No. 1886, dated 18 December 1997, the CHW activities should be guided and supervised by a nurse present in the health unit (Brazil, 1997; Brazil, 2012).

Between 1981 and 2008 there was an increase of 450% in people assisted in PHC, which helped to reduce morbidity and mortality rates. This outcome is related to the implementation of the Brazilian Family Health Program (*Programa de Saúde da Família* – PSF)^1^ in several areas where previously there was no PHC (Paim *et al*., 2011; Victora *et al*., 2011). Regarding maternal care, some Brazilian studies have shown that an adequate training program for CHWs helped to increase breastfeeding time (Victora *et al*., 2011; Pereira & Oliveira, 2013), however, there is still a need for more research regarding the roles and capacity building of the CHW in maternal/prenatal care (Orlandin *et al*., 2017).

This article presents a study of the evaluation of the knowledge of Brazilian CHWs regarding prenatal and maternal care, considering their key role in conducting health education with pregnant women.

## Methods

### Study site

This was a cross-sectional study carried out in Ribeirão Preto, a medium-size city of São Paulo state, Brazil. With ~638 thousands inhabitants (Seade, 2014), the per capita income is more than the mean of the country, being around US$412.47. The municipal human development index is 0.8 and Gini index is 0.54 (data from 2010) (Datapedia, 2013). The city has five health districts with PHC coverage of 59% (Datapedia, 2013), with the coverage of FHS teams being ~24.5% (Brazil, 2016). The city has 48 PHC units in total, of which 31 are organized according to the Traditional care model and 17 are units with FHS, employing a total of 310 CHWs in 32 health units at the time of the study.

### Study design and population

The sample size was calculated based on the estimated prevalence of knowledge regarding prenatal care by CHWs being 50% (there were no previous studies that could report on the subject), *α*=5% and a 95% confidence interval. The minimum sample size defined was 192 CHWs, which is 61.9% of the 310 CHWs employed by the municipality. Therefore, the number of CHWs to be interviewed in each PHC unit was proportional to this number, with them being selected randomly.

The questionnaire was designed based on technical documents of the Brazilian Ministry of Health, such as the ‘Technical Handbook of Prenatal Care’ (Brazil, 2000), the ‘Practical Guide for the Community Health Worker’ (Brazil, 2009a) and ‘The Work of the Community Health Worker’ (Brazil, 2009b). A draft version of the questionnaire was developed by the principal researcher and evaluated by a PHC specialist (a physician) and by two maternal health experts (nurses) who proposed some improvements. This version was administered as a pilot test with ten CHWs, who gave suggestions involving the use of words and the order of some questions.

The final version contained 75 questions, organized in three sections: sociodemographic (first), specific knowledge (second) and open-ended (third). The first had 12 questions that contemplated selected independent variables, such as age, sex, skin color, schooling, years of education, social economic classification (a national measure of economic status) [Brazilian Association of Research Companies (ABEP), 2013], years of work as a CHW, years of work in the service and whether there was an institutional link between the PHC unit and the university or not. The second section had 61 questions approaching five thematic topics, each topic had between 11 and 17 statements to be responded to as ‘right,’ ‘wrong’ or ‘unknown’. One of the topics of the second section was composed of two questions with structured answers to be completed individually or with guidance. Finally, the third section consisted of two open-ended questions, one of which asked whether the CHW was interested in learning more about any topic related to maternal health and another asking how they felt about responding to the questionnaire.

The questions of the first thematic topic of the second section of the instrument contained statements about the scope of the work of the CHW (Knowledge I). The second contained statements related to the initial approach of the pregnant woman (Knowledge II), the third was composed of statements about medical tests and vaccinations during pregnancy (Knowledge III), the fourth approached the signs and symptoms of risk in pregnancy and the signs of labor (Knowledge IV) and the final one covered general recommendation for good health during pregnancy, such as nutrition, oral health and the practice of physical exercise (Knowledge V). The original version, in Brazilian Portuguese, and an English version are in the Online Appendix.

The principal researcher met with all the managers of the PHC units with CHWs, presenting the project objectives, procedures for data collection and clarifying any doubts and questions. Then, the managers themselves, along with the researcher, presented the project to the CHWs and the selection was made through a draw. There were no refusals to participate from the managers or CHWs.

The principal researcher and two research assistants conducted the interviews. The latter receive two days of training to become familiar with the final version of the instrument, resolve any doubts and to practice using it with the former. Throughout the data collection period, the principal researcher met with the other interviewers to evaluate the quality of the data obtained and to resolve any doubts about the questionnaire application.

### Data analysis

One point was awarded for each correct response to a question of the questionnaire and zero points for wrong or unknown responses. In the Knowledge III topic, using questions with structured answers, two points were given to each spontaneous correct response, one point for guided correct responses and zero for incorrect responses. The maximum score that could be achieved was 83 and the minimum 0.

In order to analyze the score of the total knowledge and analyze and compare the scores of the specific knowledge areas, since each has a different number of questions within each block, these were standardized from 0 to 10. Based on the mean, median and the distribution of the responses of each block, the knowledge was classified into low, medium and high knowledge, or, in some cases, low and high knowledge, thus it was possible to perform the crossings and the statistical analyses.

The search for statistical associations between the independent variables and specific knowledge was performed using the *χ* ^2^ test and Fisher’s test. Statistical association was considered when *P*<0.05. The data were stored using the Epi Info software and the statistical analysis was performed using the STATA version 9 (STATA Corporation, Texas, USA) program (StataCorp, 2005).

### Ethical considerations

This study was approved by the Research Ethics Committee of the Hospital of Ribeirão Preto Medical School, University of São Paulo – USP, authorization number: 444.889.

## Results

### Sociodemographic profile

Data collection was carried out from mid November 2013 to mid January 2014 in all of the 32 health units that employed CHWs, 18 traditional health units and 14 units with FHS, with a questionnaire being administered face to face with 194 CHWs. In relation the number of CHWs, 102 were from units with FHS and 92 were from traditional health units. In the present study, 22 CHWs worked in units with FHS that were also linked to the university.

The majority of the CHWs were women (91.7%), with a mean age of 44.9 years, who self-classified themselves as being white (56.7%). The median of years of schooling found was 11.7. Almost 80% had finished high school or an equivalent course and 10% had finished a university or college course. Regarding the socioeconomic classification (A upper level and D lower level), the majority of the CHWs (69.6%) were classified as belonging to Level B (ABEP, 2013). The median time working as a CHW was 9.2 years (Table 1).

**Table 1:** Distribution of absolute and relative frequency of socio-demographic profile data found in community health worker (CHW), Ribeirão Preto, 2014

	Traditional health units		Health units with FHS		Total	
Variables	F	%	F	%	F	%
Sex						
Male	5	5.4	11	10.8	16	8.3
Female	87	94.6	91	89.2	178	91.7
Age						
20–34 years old	15	16.3	22	21.5	37	19.1
36–44 years old	35	38.0	31	30.4	66	34
45–55 years old	25	27.2	32	31.4	57	29.4
56–67 years old	17	18.5	17	16.7	34	17.5
Race/color						
White	47	51.1	63	61.8	110	56.7
Black	17	18.5	13	12.7	30	15.5
Asian	1	1.1	0	0.0	1	0.5
Mulatto	27	29.3	25	24.6	52	26.8
Indigenous color	0	0.0	1	0.9	1	0.5
Time of schooling						
4–11 years	51	55.4	56	54.9	107	55.1
12–20 years	41	44.6	46	45.1	87	44.9
Schooling						
Elementary school	9	9.8	12	11.8	21	10.8
High school	80	86.9	73	71.5	153	78.9
University or College level	3	3.3	17	16.7	20	10.3
Socioeconomic classification						
Class A	3	3.3	4	3.9	7	3.6
Class B	65	70.6	70	68.6	135	69.6
Class C	23	25	28	27.5	51	26.3
Class D	1	1.1	0	0.0	1	0.5
Time of working as CHW						
1–8 years	20	21.7	39	38.2	59	30.5
9–12 years	24	26.1	18	17.7	42	21.6
13–14 years	48	52.2	45	44.1	93	47.9
Total	92	100	102	100	194	100

FHS=Family Health Strategy.

### Total knowledge

The total score was found to be high, with a mean of 8.3, varying from 6.6 to 9.5. A total of 27.8%, scored between 6.6 and 7.9 (low), 41.7% scored from 8.0 to 8.5 (medium) and 30.5% scored from 8.6 to 9.5 (high). The distribution of the total score when standardized from 0 to 10 points showed a curve placed to the right, with the median point of 8.3. Although the CHWs presented adequate total knowledge, through each specific knowledge group, it was possible to verify the need for improvements.

### Specific knowledge

**Figure 1:**
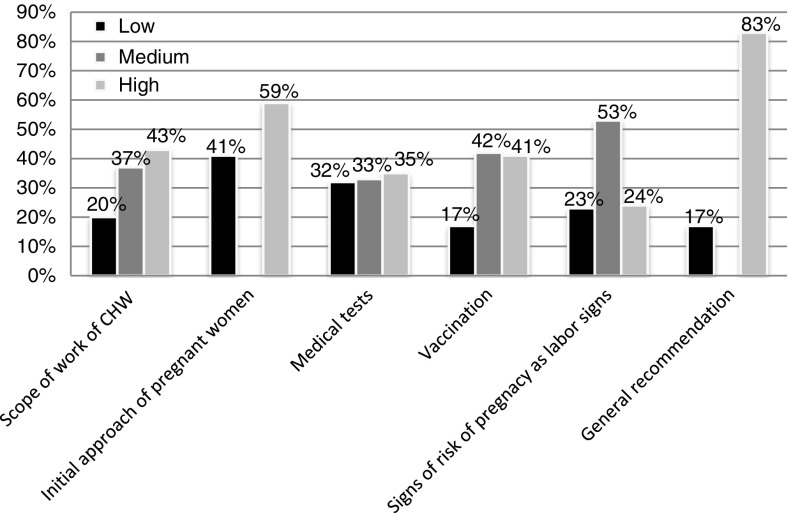
The percentage distribution of knowledge found in each group of knowledge

For each knowledge group it was possible to detect a specific score as shown in Figure 1.

Three types of knowledge were considered adequate since they included a high percentage of responses considered high or medium knowledge. These were the scope of work of CHWs (mean 9.4), the initial approach of pregnant women (mean 9.0) and general recommendation for good health of pregnant women (mean 9.0). However, the knowledge about medical tests, vaccinations and signs of risk in the pregnancy were considered insufficient, as the maximum score was achieved by 40% of the respondents, with the means being 6.9, 6.3 and 8.0, respectively. Among the medical tests more cited were blood (78%), HIV (62%), syphilis (59%), urine (56%) and diabetes (52%) tests. Among the 13 statements related to the signs of risk in pregnancy, four of them presented correct responses for a high percentage of the CHWs. These were: loss of vaginal fluid or blood, intense contractions, absence of fetal movement for more than 24 h and the presence of high fever.

### Bivariate analysis

All the independent variables (age, sex, skin color, schooling, years of education, social economic classification) were tested for association and when this was found to be positive control testing was carried out in order to avoid spurious associations. A statistically significant association was found between being female and having high knowledge about the scope of the work of CHWs (Fisher’s exact test=0.02). Being female was also found to be associated with having high knowledge about vaccinations during pregnancy (Fisher’s exact test=0.008), and about medical tests (*P*=0.03). High scores in Knowledge III – medical tests was also found to be associated with the 36–44 years age group (*P*=0.01) (Tables 2 and 3). Finally, a statistically significant association was found between being female and having high knowledge about general recommendation for good health during pregnancy (*P*=0.003), 85.4% of the female CHWs presented high knowledge, while 56.2% of the male CHWs presented high knowledge (table not shown). No other association was found among the independent and dependent variables.

**Table 2:** Distribution of the association tests among scope of work of community health worker (CHW), vaccination, medical tests and the independent variable sex of the CHW, Ribeirão Preto, 2014

	Scope of work of CHW						
	Low 11–13 pts		Medium 14 pts		High 15pts		
Variables	*N*	%	*N*	%	*N*	%	*P* ^a^
Sex							
Male	5	31.2	9	56.2	2	12.5	
Female	33	18.5	63	35.3	82	46.0	0.021

	Vaccination						
	Low 0–2 pts		Medium 3–4 pts		High 5–6 pts		
	*N*	%	*N*	%	*N*	%	*P* ^a^
Sex							
Male	7	43.7	7	43.7	2	12.5	
Female	27	15.1	74	41.5	77	43.2	0.008

	Medical tests						
	Low 6–11 pts		High 12–17 pts				
	*N*	%	*N*		%		*P* ^a^
Sex							
Male	9	56.2	7		43.7		
Female	53	29.7	125		70.2		0.03

a *P*<0.05.

**Table 3:** Distribution of the association test between medical tests and the independent variable age of the community health worker, Ribeirão Preto, 2014

	Medical tests						
	Low 6–11 pts		Medium 12–13 pts		High 14–17 pts		
AGE	*N*	%	*N*	%	*N*	%	*P* ^a^
20–35 years old	11	29.7	14	37.8	12	32.4	
36–44 years old	13	19.7	21	31.8	32	48.4	
45–55 years old	21	36.8	17	29.8	19	33.3	0.019
56–67 years old	17	50.0	12	35.2	5	14.7	

a *P*<0.05.

The sex variable was found to be associated with length of time working in the service, with the female CHWs having been working longer in the service than the male CHWs (Fisher’s exact test <0.001). The age variable was found to be associated with years of schooling. The CHWs that were in the 36–44 years age group presented greater knowledge about medical tests than the other groups in the controlled test (Fisher’s exact test=0.032) and had a greater length of schooling (*P*=0.04) (tables not shown). Statistical analyses were performed comparing the knowledge between the CHWs of the units linked to the university and the other CHWs and also between the CHWs of the traditional health units and those of the health units with FHS and no statistically significant differences were found in either of the two comparisons.

Regarding the final part of the questionnaire with two open questions, in the first there were 279 responses suggesting themes of interest. These responses were given by 143 CHWs (73.7%), while 51 CHWs (26.3%) said they had no interest in any topic related to pregnancy. The 279 suggested themes which were topics highlighted for training were: vaccinations (13.6%), oral health (7.8%), examinations recommended during pregnancy (6.1%), diseases associated with pregnancy or that may occur, such as diabetes, gestational hypertension, preeclampsia, ectopic gestation, toxoplasmosis and syphilis, and signs of risk (4.3%) and signs of labor (4.3%).

In the second question on how they felt about answering the questionnaire the 194 CHWs described 292 types of feelings. Regarding these 292 answers, some of the CHWs (30.5%) reported feeling that ‘it was easy to answer the questionnaire’ and the majority of the answers (92.1%) were related to a positive experience or feelings such as: ‘it helped to evaluate the knowledge,’ ‘it highlighted the need to study more,’ ‘it was good,’ ‘important,’ ‘fast,’ ‘it renewed knowledge,’ ‘I felt comfortable’ and ‘it helped to valorize the work of the CHW.’ Only 23 responses (7.8%) reported any negative experiences, such as ‘anxiety,’ ‘doubts,’ ‘tension’ and ‘shame.’

Considering the repercussion of the study, the regional health authority showed interest in the results, which generated technical training with nurses, not only in the municipality but also in other municipalities, as knowledge multipliers for CHWs. Authorization and collaboration was requested from the researcher to use the instrument to assess the knowledge of CHWs in other cities in the region, detecting training needs in each location.

## Discussion

CHWs can play an important role in the improvement of the quality of prenatal care in Brazil, since the country did not achieve The Millennium Goal of decreasing the maternal mortality rate to 35 deaths per 100 000 births by the year 2015 (Souza, 2013). Although there has been some reduction, the latest data from the WHO showed that the maternal mortality rate in 2015 was 44 deaths per 100 000 live births, therefore, the reduction of maternal mortality in the country is still a challenge (WHO, 2015). The new goals established for the year 2030 still include the reduction of the maternal mortality rate, which is at the center of the agenda of the Sustainable Development Goals over the next 15 years (Souza, 2015; United Nations, 2015b).

The demographic profile of the CHWs was similar to the findings of other studies conducted in Brazil, showing that the majority of the CHWs were female and the greatest proportion of them had concluded high school (Moura *et al*., 2010; Mascarenhas *et al*., 2013; Rocha *et al*., 2015). Some other studies conducted in Brazil have shown similar results to those of the present study regarding age groups, sex and length of schooling ( Barcellos *et al*., 2006; Moura *et al*., 2010; Santos *et al*., 2011; Barbosa *et al*., 2012). Considering the fact that the majority of CHWs are female, Barbosa *et al*. (2012) highlighted that the choice of this profession is probably related to gender roles in society and the feminine role of caring.

Although the total score showed that the CHWs had good knowledge regarding the scope of their work, some specific areas of knowledge related to prenatal care were identified as being in need of training and capacity building, as shown by the specific knowledge scores, such as medical tests, vaccinations, the signs and symptoms of risk in pregnancy and signs of labor. The CHW as a professional career is very recent in Brazil (regulated legally in 2002 Brazil, 2006), with a wide range in the level of schooling permitted due to the social inequality in the country. However, the municipality where the research was conducted has a high Human Developed Index and this could have influenced the level of general knowledge shown by the CHWs. Even so, the questionnaire was effective in determining some specific gaps in the knowledge. It is important to note that CHWs in Brazil receive local training on topics related to PHC and the general work as a CHW before starting the work, however, not necessarily on specific themes, such as conditions or diseases. This can be observed by the high knowledge presented by the majority of the CHWs in the first block which is about the work of the CHW.

No articles specifically describing the assessment of the prenatal care knowledge of CHWs were found in the international literature. Thus, the results obtained with the CHWs in the present study were compared with other studies that evaluated the knowledge of pregnant women about the topics. The study of Mendoza-Sassi *et al*. (2007) assessed the knowledge of pregnant women regarding prenatal care and pregnancy risk situations. Considering the self-reported responses of 367 women interviewed about medical tests, 81% cited blood tests; 66% mentioned urine and HIV tests; 33% cited ultrasound examinations; and between 15% and 17% tests for the detection of diabetes, syphilis and hepatitis. Only 6% cited the Pap test for cervical exams as being recommended for all pregnant women. The results of the present study corroborated the findings of Mendoza-Sassi *et al*. (2007) in the majority of the medical tests except for urine and syphilis tests, in which the CHWs achieved better results.

A difference related to the knowledge of vaccinations was found when comparing the study results with those of the study conducted by Healy *et al*. (2015) in the United States. The authors showed that 84% of the pregnant women knew the vaccinations recommended during pregnancy. These results are probably related to education or cultural differences between women from the two countries, since in this sample the majority of the pregnant women 52.1% had, at least, a university degree and only 15.2% of the sample had an education level of completed high school or less.

Regarding the knowledge of signs of risk in the pregnancy and signs of labor the findings contrast with the results of Mendoza-Sassi *et al*. (2007). The authors revealed that 66% of the women reported that vaginal bleeding and severe abdominal pain or strong contraction are warning signs and only one in ten said that the cessation of fetal movement indicated severity during pregnancy needing immediate medical evaluation. The authors also showed that a significant percentage of the pregnant women did not recognize some of these situations as risks for the pregnancy, indicating poor knowledge.

Based on the study findings, female CHWs showed greater knowledge than male CHWs in relation to the medical tests and vaccinations. Furthermore, the 36–44 years age group demonstrated high levels of knowledge. This age group also had a greater length of schooling (12–20 years). A hypothesis is that this age group coincides with the end of the reproductive phase, that is, pregnancy is a recent memory, as are the recommended tests. Specifically regarding vaccinations, the greater knowledge of the women could be explained due to the women presenting more time working as CHWs in relation to the men.

A limitation of the study is related to the fact that the questionnaire was not tested with other population types, such as other health providers. Another limitation is the possible use of this questionnaire in the future in other countries and realities, since in each country or region the CHWs can have attributes that are different to the Brazilian technical norms on pregnancy used in this instrument. This means that to use this questionnaire in another country it will be necessary to adapt it according to the specific context. However, the strength of the study was in identifying the specific problematic areas with the need for training of the CHWs for prenatal care knowledge. Furthermore, the assessment was completely based on the recommendation of the Brazilian Health Ministry and the technical guide for CHW practice.

## Conclusion

The CHW has the potential to support and improve prenatal care. To assess the knowledge of CHWs regarding the prenatal care program is an important step in deciding the contents of training programs. The problematic areas identified as having a need for training were medical tests, vaccinations and signs and symptoms of risk and signs of labor, therefore, from these results, a training plan was designed and a training intervention was implemented in the region.

As the questionnaire was entirely based on the knowledge of technical standards of the Ministry of Health, it can be used in other places and the construction of this instrument may, in the future, serve as a model for developing tools to assess other issues related to maternal health or the scope of the work of the CHW, as well as that of other health providers, and to evaluate their knowledge regarding the subject.

## Footnotes

1
Currently the Family Health Program in Brazil is named the Family Health Strategy (FHS) and from now on this terminology will be used.
